# Retinal microvasculature and imaging markers of brain frailty in normal aging adults

**DOI:** 10.3389/fnagi.2022.945964

**Published:** 2022-08-22

**Authors:** Wendan Tao, William Robert Kwapong, Jianyang Xie, Zetao Wang, Xiaonan Guo, Junfeng Liu, Chen Ye, Bo Wu, Yitian Zhao, Ming Liu

**Affiliations:** ^1^Department of Neurology, West China Hospital, Sichuan University, Chengdu, China; ^2^The Affiliated People’s Hospital of Ningbo University, Ningbo, China; ^3^Cixi Institute of Biomedical Engineering, Ningbo Institute of Materials Technology and Engineering, Chinese Academy of Sciences, Ningbo, China; ^4^Department of Radiology, West China Hospital, Sichuan University, Chengdu, China; ^5^School of Information Science and Engineering, Yanshan University, Qinhuangdao, China

**Keywords:** cerebral small vessel disease, brain frailty, brain volume, retinal microvasculature, swept-source optical coherence tomography

## Abstract

**Background:**

The retina and brain share a similar embryologic origin, blood barriers, and microvasculature features. Thus, retinal imaging has been of interest in the aging population to help in the early detection of brain disorders. Imaging evaluation of brain frailty, including brain atrophy and markers of cerebral small vessel disease (CSVD), could reflect brain health in normal aging, but is costly and time-consuming. In this study, we aimed to evaluate the retinal microvasculature and its association with radiological indicators of brain frailty in normal aging adults.

**Methods:**

Swept-source optical coherence tomography angiography (SS-OCTA) and 3T-MRI brain scanning were performed on normal aging adults (aged ≥ 50 years). Using a deep learning algorithm, microvascular tortuosity (VT) and fractal dimension parameter (D_box_) were used to evaluate the superficial vascular complex (SVC) and deep vascular complex (DVC) of the retina. MRI markers of brain frailty include brain volumetric measures and CSVD markers that were assessed.

**Results:**

Of the 139 normal aging individuals included, the mean age was 59.43 ± 7.31 years, and 64.0% (*n* = 89) of the participants were females. After adjustment of age, sex, and vascular risk factors, D_box_ in the DVC showed a significant association with the presence of lacunes (β = 0.58, *p* = 0.007), while VT in the SVC significantly correlated with the score of cerebral deep white matter hyperintensity (β = 0.31, *p* = 0.027). No correlations were found between brain volumes and retinal microvasculature changes (*P* > 0.05).

**Conclusion:**

Our report suggests that imaging of the retinal microvasculature may give clues to brain frailty in the aging population.

## Introduction

Brain frailty is usually recognized as the product of physiologic changes associated with advancing age and the accumulation of multiple diseases (Yassi et al., 2020). Evidence showed that it would cause a loss of resilience to acute health problems, which may result from both the diseases and extrinsic stressors (Bu et al., 2021). Brain frailty is detected on neuroimaging or postmortem as atrophy and markers of cerebral small vessel disease (CSVD), including white matter hyperintensities (WMHs), lacunes, enlarged perivascular spaces (EPVSs), lacunar infarcts, and microbleeds (Tschirret et al., 2018; Appleton et al., 2020). These brain parenchyma lesions can cause an acute stroke syndrome known as lacunar stroke, or more subtle pathological alteration, which may eventually lead to neurological deficits and cognitive decline in the long term. In the aging population, the absence of CSVD and the presence of large brain volumes are imaging markers of brain health (Gardener et al., 2018; Pase et al., 2018). Although neuroimaging is sensitive to brain frailty, it is costly, time-consuming, and hardly used as a screening tool for a healthy brain; thus, simpler, inexpensive, and reproducible biomarkers that can be used on a large population would offer the prospect of enabling earlier evaluation of brain global health.

The retina shares embryologic origin and microvascular characteristics with the brain and is widely regarded as part of the central nervous system (Chang et al., 2014; Hart et al., 2016). Retinal thickness and microvasculature offer a unique route to evaluate tissues that are associated with the cerebral structures suggesting a clear link between retinal and cerebral changes (Mutlu et al., 2016, 2017; McGrory et al., 2017; Méndez-Gómez et al., 2018). Consequently, the retina is vulnerable to similar neurodegeneration processes that are associated with cerebral small vessel disease (CSVD) features and cerebral atrophy.

Previous reports using fundus photography showed that retinal vessels are associated with radiological indicators of CSVD in the aging population (Cheung et al., 2010b; Ballerini et al., 2020); however, fundus photography cannot give information on the deeper microvasculature. Accumulating reports using optical coherence tomography (OCT) have shown that retinal structure is associated with cerebral microstructural changes and radiological indicators of CSVD in the aging population (McGrory et al., 2019; Chua et al., 2021). OCT angiography (OCTA) is an extension of the OCT that non-invasively images the retinal microvasculature at a high resolution; this imaging modality provides in-depth information and visualization of the retinal microvasculature in different retinal layers (i.e., the superficial and deep microvasculature). To date, retinal assessment in the aging population was mainly confined to conventional angiography, which cannot give information on the deeper retinal microvasculature.

The recent development of new quantitative OCTA indicators provided a step forward in evaluating the retinal microvasculature features such as fractal dimension (Dbox) and microvascular tortuosity (VT) (Lemmens et al., 2020); these features are considered an indirect measure of blood flow in the retinal microvasculature. To the best of our knowledge, these parameters have never been used in reports focusing on the aging population.

Our current study employed the swept-source optical coherence tomography angiography (SS-OCTA) and evaluated microvascular changes in the retina and its correlation with frailty imaging markers assessed on MRI in an aging volunteer cohort.

## Materials and methods

### Study population

Neurologically normal individuals (aged ≥ 50 years) were recruited as part of a healthy aging study from the Neurology Department at West China Hospital, China. Inclusion criteria included neurologic and neuropsychological examination with normative standards as previously reported (Casaletto et al., 2017), no major memory concerns or a diagnosed memory condition, and the capability to independently complete activities of daily living by a clinical dementia rating of 0 (Duff et al., 2022). Participants were additionally screened for sarcopenia by the SARC-F questionnaire (5 components: Strength, Assistance with walking, Rise from a chair, Climbing stairs, and Falls) (Malmstrom and Morley, 2013). The SARC-F scores ≥ 4 are predictive of sarcopenia, which could potentially indicate an unhealthy state and will not be included. In addition, individuals who were diagnosed with mental diseases, including depression or taking antipsychotics, were also not included.

Additionally, individuals were screened for ophthalmic conditions that could potentially impact the structure and microvasculature of the retina such as diabetic retinopathy, preexisting glaucoma, cataract, age-related macular degeneration, optic neuritis, and high myopia.

Participants answered a wide-ranging questionnaire covering demographic, education, and self-reported vascular risk factors, including hypertension, diabetes, smoking, and alcohol consumption information. Cognitive measures included the Mini-Mental State Examination (MMSE) and the Montreal Cognitive Assessment (MoCA-BJ) (Yu et al., 2012).

The Medical Ethics Committee of West China Hospital, Sichuan University approved the study under the principles of the Declaration of Helsinki (2020–104). Written informed consent was obtained from every participant.

### Brain image acquisition and volumetric measures of brain structure

Image acquisition was performed using a standard 3T scanner (Siemens Skyra) with a 32-channel head coil at West China Hospital of Sichuan University. Sequences consisted of T1- and T2-weighted imaging, fluid-attenuated inversion recovery (FLAIR), and susceptibility-weighted imaging (SWI). T1-weighted high-resolution images were acquired by a 3D magnetization-prepared rapid gradient-echo (MPRAGE). Imaging parameters were repetition time (TR) = 1,900 ms; echo time (TE) = 2.4 ms; FA = 9°; field of view (FOV) = 250 mm; 256 × 192 matrix; 191 slices; and voxel dimension = 1.0 mm × 1.0 mm × 1.0 mm.

T1-weighted structural images were processed using Computational Anatomy Toolbox 12 (CAT12)^1^ for Statistical Parametric Mapping (SPM) 12 (Wellcome Trust Center for Neuroimaging, London, United Kingdom). Each structural image was first visually inspected for artifacts and then manually reoriented to set the image origin at the anterior commissure. The reoriented images were spatially normalized to the Montreal Neurological Institute space and segmented into gray matter (GM), white matter (WM), and cerebrospinal fluid, using the standard tissue probability maps provided in SPM12. Volume changes induced by normalization were adjusted using the Jacobian modulation. Spatially normalized GM images were finally smoothed using a Gaussian kernel with a full width at half a maximum of 8 mm. Total intracranial volumes (TIVs) were calculated by summing the volume values of the gray matter, white matter, and cerebrospinal fluid. Bilateral hippocampus volumes were calculated using the automated anatomical labeling (AAL) template.

### Cerebral small vessel disease magnetic resonance imaging markers rating

Small vessel disease MRI markers of lacunes, white matter hyperintensity (WMH), cerebral microbleeds (CMBs), and enlarged perivascular spaces (EPVSs) were rated according to the STandards for Reporting Vascular changes on nEuroimaging (STRIVE) consensus criteria (Wardlaw et al., 2013).

Lacunes were defined as rounded or ovoid lesions involving the subcortical regions, 3–15 mm in diameter, of CSF signal intensity on T2 and FLAIR, generally with a hyperintense rim on FLAIR and no increased signal on DWI. WMH was defined as a high signal intensity region on the FLAIR sequence. The extent of periventricular and deep WMH was rated using the Fazekas scale where periventricular WMH extends into the deep white matter (Fazekas score 3) or deep WMH (Fazekas score 2 or 3) was regarded as severe WMH. CMBs were defined as homogeneous rounded hypointense lesions on susceptibility-weighted imaging with a diameter of 2–10 mm. EPVSs were defined as small (< 3 mm) round or linear hyperintense lesions on T2-weighted images in the basal ganglia or centrum semiovale and rated as 0 to 4 on a validated semiquantitative scale. We only counted EPVS in the region of the basal ganglia, which were specifically identified to be associated with CSVD. An ordinal score ranging from 0 to 4 was constructed to reflect the total burden of CSVD, as previously described. The interobserver agreement of measurements for each CSVD neuroimaging marker (lacunes, WMH, CMBs, and EPVS) was considered good to excellent.

Magnetic resonance imaging images were visually inspected with software (RadiAnt DICOM Viewer1.0.4.4439; Medixant Ltd., Poznan, Poland) and evaluated by a single rater (TWD) blind to clinical information and OCT data. A second rater (YC) evaluated a random sample of 20 patients to assess inter-rater agreement for the presence of lacunes (kappa 0.83, *P* < 0.001), EPVS in CSO (kappa 0.65, *P* < 0.001), EPVS in BG (kappa 0.75, *P* < 0.001), the severity of WMH (kappa 0.70, *P* < 0.001), and presence of microbleeds (kappa 0.85, *P* < 0.001).

### Retinal microvascular imaging with swept-source optical coherence tomography angiography

**FIGURE 1:**
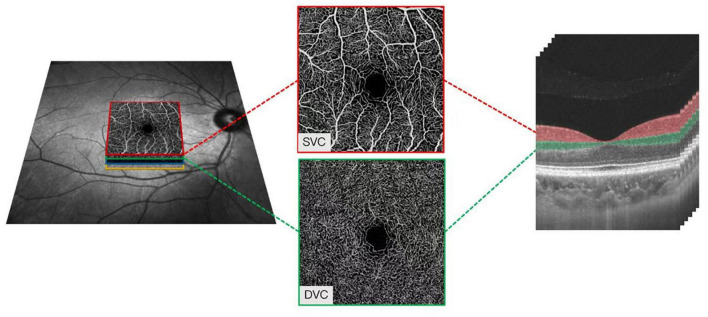
Illustrative image of the macular microvasculature. The superficial vascular plexus (SVC) and deep vascular plexus (DVC), which was 5 μm above the inner limiting membrane (ILM) to 25 μm below the lower layer of the inner nuclear layer (INL).

With a central wavelength of 1,050 nm and a scan rate of 200,000 A-scan per second, the SS-OCTA, which contained a swept-source laser, was used to image the retinal microvasculature of all the participants. The tool was set with an eye-tracking function based on an integrated confocal scanning laser ophthalmoscope to remove eye-motion artifacts. The lateral resolution, axial resolution, and scan depth were 13 μm, 5 μm, and 3 mm, respectively. Software in the tool segmented the retinal microvasculature into the superficial vascular plexus (SVC) and deep vascular plexus (DVC), which was 5 μm above the inner limiting membrane (ILM) to 25 μm below the lower layer of the inner nuclear layer (INL), as shown in Figure 1.

The quality of the macular images was assessed objectively and subjectively, rejecting images with a signal quality less than 7 on a scale of 10. Participants who could not cooperate during imaging were excluded from our data analysis (severe eye movement and head movement during imaging could produce artifacts that could affect the data). Angiograms with retinal diseases such as optic neuritis and age-macular degeneration were excluded from data analysis.

### Quantification of the macular microvasculature based on deep learning

The OCTA-Net was utilized for microvasculature segmentation. This model consists of a split-based coarse segmentation and a split-based refining segmentation module, to produce a preliminary confidence map, and optimize the contour of the retinal microvasculature, respectively (Ma et al., 2021). The OCTA-Net was trained on a public OCTA dataset named ROSE-1, and its efficiency has been validated; the results had a good performance [the area under the curve (AUC) = 0.9505, ACC = 0.9235, G-mean = 0.8374, Kappa = 0.7349, Dice = 0.7808, and false discovery rate (FDR) = 0.1478] when compared to the ground truth (GT).

**FIGURE 2:**
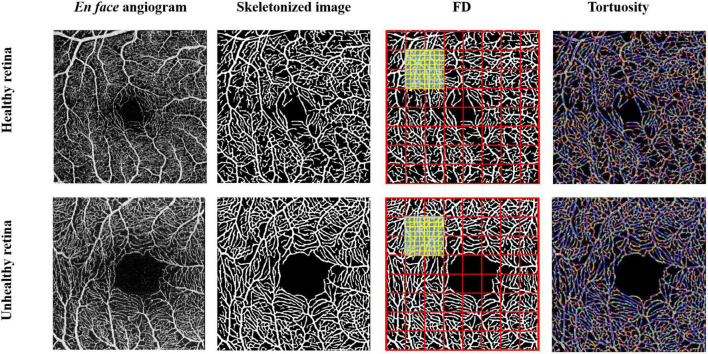
Quantification of macular microvasculature of a healthy and unhealthy retina using the deep-learning algorithm. Enface images of a healthy and unhealthy retina from the swept-source optical coherence tomography angiography (SS-OCTA) were converted into a skeletonized image. Fractal dimensions using D_box_ and microvascular tortuosity were extracted from the skeletonized image using MATLAB.

In brief, OCTA images in PNG format were exported to a custom-built algorithm software. Images were segmented to obtain the microvasculature in the SVC and DVC. The images were then skeletonized and the foveal avascular zone (FAZ) was extracted. The microvascular tortuosity (VT) and fractal dimension using D_box_ were calculated based on the segmentation map using MATLAB as previously reported (Zhao et al., 2020). Microvascular tortuosity is a metric to measure the tortuous level of the vasculature and is calculated by applying the method proposed by Zhao et al. (2020). Vascular fractal dimension is a well-known measure of the geometric complexity of vasculature and is calculated according to the algorithm proposed in a previous report (Ma et al., 2021). Figure 2 shows the quantification of the microvasculature based on deep learning.

### Statistical analysis

Continuous variables with normal distribution were expressed as mean ± SD, while skewed distribution was expressed as medians and interquartile ranges. Categorical variables are presented as frequencies and percentages. The z-scores of all the SS-OCT/SS-OCTA parameters and brain MRI volumes were calculated by subtracting the mean value from the value of the observation and dividing by the SD. The univariate analysis (Pearson’s or point-biserial correlation) was used to examine the association between vascular risk factors (hypertension, diabetes mellitus, hyperlipidemia, present drinker, and present smokers) and CSVD markers and brain MRI volumes. Variables with *P* < 0.1 in the univariate analysis and factors commonly considered as confounders were included in the multivariate analysis. The multivariate linear regression based on a generalized estimating equation was used to investigate the association between SS-OCTA parameters and neuroimaging parameters. We additionally adjusted for education years when the outcome was the cognitive tools (MoCA and MMSE). The ß coefficients represent a standardized mean difference in z-scores of total brain, gray and white matter, and hippocampal, per SD decreased in retinal vascular FD and increased tortuosity normalized. All the analyses were performed with SPSS (version 24, SPSS Incorporation.). *P*-values less than 0.05 were considered statistically significant.

## Results

We initially enrolled 160 neurologically normal individuals who were dementia- and stroke-free. Out of the participants, 4 participants could not cooperate during MRI and were excluded. Of the 156 individuals who underwent SS-OCTA imaging, 3 individuals could not cooperate, 8 individuals had severe cataracts, and 6 individuals had poor imaging signal quality (presence of artifacts on angiograms due to eye movement or head movement during imaging).

Our study finally included 139 participants who had their MRI scans and SS-OCTA angiograms, as well as baseline information as shown in Table 1. The mean age was 59.43 ± 7.31 years, and 64.0% (*n* = 89) of the participants were females. Of 139 normal participants, 27 (19.4%) participants had hypertension, 5 (3.6%) participants had diabetes mellitus, and 18 (12.9%) participants had dyslipidemia. The mean educational level was 11.32 ± 4.18 years. The median MMSE score was 28 and the MoCA score was 25.

**TABLE 1:** Clinical and neuroimaging information of study participants.

Characteristics	Descriptive
Age, y	59.43 ± 7.31
Female	89 (64.0)
Education, y	12 (9–16)
Hypertension	27 (19.4)
Diabetes mellitus	5 (3.6)
Dyslipidemia	18 (12.9)
Present smokers	21 (15.1)
Present drinkers	32 (23.0)
MMSE	28 (27–30)
MoCA	25 (23–28)
Presence of lacunes	6 (4.3)
PWMH	1.0 (0–1.0)
DWMH	1 (1.0–1.0)
Presence of Microbleeds	16 (11.5)
BG-EPVS	1.0 (0–1.0)
CSO-EPVS	2 (1.0–2.0)
Total brain volume,	1386.99 ± 127.05
White matter volume	491.84 ± 55.18
Gray matter volume	596.82 ± 43.48
Hippocampus volume	3.85±0.34
VT in SVC	1.57±0.16
D_box_ in SVC	1.63±0.03
VT in DVC	1.30±0.30
D_box_ in SVC	1.70±0.01

Data are n (%), mean (SD) or median (IQR).

MMSE, mini-mental state examination; MoCA, Montreal Cognitive Assessment; PWMH, periventricular white matter hyperintensity; DWMH, deep white matter hyperintensity; BG-EPVS, basal ganglia-enlarged perivascular spaces; CSO-EPVS, centrum semiovale-enlarged perivascular spaces; SVC, superficial vascular complex; DVC, deep vascular complex; VT, microvascular tortuosity; D_box_, fractal dimension.

Taking into account the influence of vascular risk factors on SVD markers and brain volume change, the univariate analyses showed hypertension significantly correlated with more severe total WMH (*r* = 0.14, *p* = 0.095) and EPVS (BG, *r* = 0.20, *p* = 0.017; CSO, *r* = 0.14, *p* = 0.099), while diabetes associated with the presence of lacunes (*r* = 0.34, *p* < 0.001) and total WMH (*r* = 0.15, *p* = 0.077). In addition, present drinkers and smoker correlated with brain volumetric measures (Supplementary Table 1). Thus, hypertension, diabetes, present smokers, and drinkers were introduced as independent variables in multivariate models of analysis. Age, sex, and dyslipidemia were additionally adjusted and considered as commonly confounding factors. In the multivariate analysis, no significant associations were found between brain MRI volumetric measures and retina microvasculature changes.

### Association between macular microvasculature and brain imaging markers of cerebral small vessel disease

D_box_ in the DVC showed a significant association with the presence of lacunes (β = 0.58, *p* = 0.007, Table 2). Microvascular tortuosity (VT) in the SVC significantly correlated with the score of cerebral deep WMH (β = 0.31, *p* = 0.027, Table 2).

**TABLE 2:** Correlation between MRI parameters and OCTA measures.

	SVC				DVC			
Variable	VT		D_box_		VT		D_box_	
	β Coefficient	*P* value	β Coefficient	*P* value	β Coefficient	*P* value	β Coefficient	*P* value
	(95% CI)		(95% CI)		(95% CI)		(95% CI)	
Total brain	0.02 (–0.19 to 0.22)	0.881	−*0*.*002*(−*0*.*008* to *0*.*004*)	0.550	0.09 (–0.12 to 0.31)	0.406	0.02 (–0.15 to 0.19)	0.817
White matter	−*0*.*007*(−*0*.*20* to *0*.*19*)	0.943	0.01 (–0.15 to 0.17)	0.873	0.05 (–0.13 to 0.24)	0.553	−*0*.*03*(−*0*.*18* to *0*.*13*)	0.749
Gray matter	−*0*.*03*(−*0*.*20* to *0*.*14*)	0.735	0.10 (–0.05 to 0.24)	0.19	−*0*.*04*(−*0*.*25* to *0*.*17*)	0.705	−*0*.*07*(−*0*.*20* to *0*.*06*)	0.301
Hippocampus 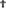	−*0*.*08*(−*0*.*26* to *0*.*10*)	0.385	0.03 (–0.14 to 0.21)	0.721	0.08 (–0.09 to 0.25)	0.386	−*0*.*03*(−*0*.*18* to *0*.*12*)	0.706
**CSVD markers**								
Lacunes	0.47 (–0.26 to 1.20)	0.208	0.20 (–0.12 to 0.52)	0.222	−*0*.*15*(−*0*.*93* to *0*.*63*)	0.703	0.58 (0.15 to 0.93)	0.007
PWMH	0.13 (–0.10 to 0.36)	0.274	0.20 (–0.04 to 0.44)	0.107	−*0*.*12*(−*0*.*37* to *0*.*16*)	0.419	0.04 (–0.16 to 0.24)	0.683
DWMH	0.31 (0.03 to 0.58)	0.027	0.02 (–0.32 to 0.36)	0.928	−*0*.*14*(−*0*.*42* to *0*.*15*)	0.350	−*0*.*06*(−*0*.*30* to *0*.*19*)	0.640
Microbleeds	0.20 (–0.21 to 0.61)	0.343	−*0*.*11*(−*0*.*58* to *0*.*36*)	0.647	−*0*.*21*(−*0*.*82* to *0*.*40*)	0.496	−*0*.*11*(−*0*.*50* to *0*.*28*)	0.581
BG-EPVS	0.04 (–0.21 to 0.28)	0.774	−*0*.*13*(−*0*.*32* to *0*.*05*)	0.162	0.0 (–0.24 to 0.25)	0.983	0.12 (–0.07 to 0.32)	0.221
CSO-EPVS	0.09 (–0.11 to 0.29)	0.385	0.05 (–0.10 to 0.19)	0.529	−*0*.*14*(−*0*.*34* to *0*.*07*)	0.196	0.02 (–0.13 to 0.17)	0.794
MoCA 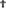	−*0*.*06*(−*0*.*11* to −*0*.*02*)	0.011	−*0*.*05*(−*0*.*09* to *0*.*0002*)	0.051	0.05 (–0.013 to 0.104)	0.128	−*0*.*05*(−*0*.*09* to *0*.*001*)	0.055
MMSE 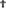	−*0*.*08*(−*0*.*17* to *0*.*005*)	0.064	−*0*.*02*(−*0*.*11* to *0*.*08*)	0.759	0.02 (–0.08 to 0.12)	0.702	−*0*.*03*(−*0*.*11* to *0*.*06*)	0.575

Values represent standardized mean difference in z-scores of brain MRI volumetric measures (95% confidence interval) per SD decrease in average SVD and DVC tortuosity and D_box_ change.

Values are adjusted for age, sex, hypertension, diabetes mellitus, hyperlipidemia, alcohol intake, and current smokers.

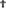
Additional adjusted for education.

PWMH, periventricular white matter hyperintensity; DWMH, deep white matter hyperintensity BG-EPVS, basal ganglia-enlarged perivascular spaces; CSO-EPVS, centrum semiovale-enlarged perivascular spaces; MMSE, mini-mental state examination; MoCA, Montreal Cognitive Assessment; SVC, superficial vascular complex; DVC, deep vascular complex; VT, microvascular tortuosity; D_box_, fractal dimension parameter.

### Association between macular microvasculature and cognitive tools

The MoCA scores significantly correlated with microvascular tortuosity in the SVC (β = −0.06, *p* = 0.011, Table 2). In addition, we could find a marginal significance between MoCA and D_box_ in the SVC (β = −0.05, *p* = 0.051) and DVC (β = −0.05, *p* = 0.055).

## Discussion

Magnetic resonance imaging for clinical indications is becoming frequent and radiological frailty indicators of CSVD and brain atrophy are commonly reported incidental findings, especially in aging individuals. WMH and lacunes of presumed vascular origin are recognized as radiological indicators of SVD. Our study shows that deep WMH presumed of vascular origin correlated with retinal tortuosity in SVC, while fractal dimension values in the DVC correlated with the presence of lacunes. Importantly, our results showed that SVC tortuosity significantly correlated with the MoCA scores. Taken together, these results suggest that macular microvascular changes may reflect the cerebral radiological indicators associated with brain frailty in aging individuals.

Microvascular tortuosity is suggested as a measure of blood flow (Vilela et al., 2021). Our current report showed that tortuosity in the SVC correlated with deep WMH as measured with the Fazekas scale in our aging population. Increased microvascular tortuosity is reflective of dysfunction of the microvascular wall, disturbed blood flow, tissue hypoxia, and dysfunction of the blood–retina barrier (Cheung et al., 2012; Tapp et al., 2019). Related processes have been detailed to result in cerebral WMH proposing that similar mechanisms on the microvascular level occur concurrently in the retina and the brain. On the other hand, increased macular microvasculature tortuosity has been linked with hypertension, cognitive impairment, and stroke and is suggested to represent microvascular impairment due to the fall of the vessels (Cheung et al., 2013; Ong et al., 2013; Tapp et al., 2019; Wu et al., 2020). Deep WMH reflects microvascular damage in the brain and is associated with CSVD, thus we suggest that microvascular changes in the SVC may be associated with cerebral microvascular changes, which are in line with previous reports (Ikram et al., 2006; Doubal et al., 2010a; McGrory et al., 2019).

The fractal dimension describes the complexity of the branching pattern and the density of the vascular system. Accumulating retinal imaging reports have shown that reduced fractal dimension in the retina is linked with hypertension, ischemic stroke, and diabetes and is thought to reflect microvascular impairment due to the destruction of the microvasculature, thus creating a simpler microvascular network (Doubal et al., 2010b; Frydkjaer-Olsen et al., 2015; Kostic et al., 2018; McGrory et al., 2019; Lemmens et al., 2020; Rim et al., 2020). Previous reports using retinal photographs showed that retinal microvascular abnormalities are associated with subclinical cerebral infarction (Cheung et al., 2010a,b; Ikram et al., 2013). Our current report showed that fractal dimension in the DVC significantly correlated with the presence of lacunes of presumed vascular origin. Lacunes are an expression of cerebral ischemia (microvascular damage) from different etiologies and are also suggested as a radiological marker of CSVD. DVC consists of capillaries (responsible for the diffusion of oxygen) and is sensitive to the ischemic changes in the retina (Wang et al., 2018). The correlation between the DVC and the presence of lacunes of presumed origin suggests that ischemic changes in the deeper retinal microvasculature reflect the ischemic changes in the brain.

Neuropsychological assessments such as the MoCA is a fundamental approach that is used to assess the cognition in individuals. The association between OCTA variables and the MoCA has been suggested to be useful in the clinical evaluation and monitoring of patients with cognitive dysfunction (Zhang et al., 2020; López-Cuenca et al., 2021). Our current report showed that the MoCA scores significantly correlated with SVC changes in aging individuals, which are congruent with previous reports. The significant association of the MoCA and SVC gives meaningful evidence for OCTA investigation for cognitive screening.

Our study has some limitations. The average age in our volunteer cohort was younger than in other similar studies (Ikram et al., 2006; McGrory et al., 2019; Chua et al., 2021). This might be an important reason why we did not find a significant relationship between markers of cerebral atrophy and retinal neurodegeneration. Another limitation is that the participants included in our study did not have eye-related disorders or brain disorders, resulting in the inclusion of relatively normal individuals, which might have caused the underestimation of our report. In addition, as a cross-section design, we cannot verify the cause and effect of the relationship between retinal microvasculature and brain abnormality, and the potential mechanisms need further investigation. The OCTA imaging procedure requires concentration and cooperation from individuals, which makes some of the images obtained inappropriate for analysis; head movement, constant eye blinking, and eye movement during imaging produce artifacts that may affect data.

In conclusion, this study represents the first OCTA study investigating the retinal microvasculature based on a deep learning algorithm and its relationship with CSVD markers in the aging population. As the main findings, we showed that SVC correlated with deep WMH, while DVC correlated with the presence of lacunes of presumed vascular origin; we also showed that SVC correlated with the MoCA scores in the aging population. Our study suggests that OCTA could be used as a potential marker of CSVD indicators and initiate extensive cognitive evaluation and reliable monitoring in the aging population. Future studies with larger sample sizes and longitudinal study designs will be needed to validate the suitability of macular microvasculature assessment with OCTA monitoring as an imaging biomarker of CSVD and cognition in the aging population.

## Data availability statement

The raw data the conclusions of this article will be made available by the authors, without undue reservation.

## Ethics statement

The studies involving human participants were reviewed and approved by The Medical Ethics Committee of West China Hospital, Sichuan University approved the study under the principles of the Declaration of Helsinki (2020-104). The patients/participants provided their written informed consent to participate in this study.

## Author contributions

WT: study concept, individual recruit, data analysis, statistics, and article writing. WK: article writing, OCT imaging scanning, and data analysis. JX and YZ: OCT imaging data analysis. ZW: brain MRI imaging scanning. XG: MRI imaging data analysis. JL: individual recruit. CY: individual recruit and CSVD markers reading. BW: study guidance. ML: study concept and guidance. All authors have reviewed and approved the submitted version of the manuscript.

## Footnotes

1
http://dbm.neuro.uni-jena.de/cat/
