# Supplementary material for: Retinal microvasculature and imaging markers of brain frailty in normal aging adults
Source: Front Aging Neurosci. 2022 Aug 22;14:945964. doi: 10.3389/fnagi.2022.945964 (PMC9441884; doi:10.3389/fnagi.2022.945964)
Supplement: Supplementary file 1 [file Table_1.docx]

**Supplementary Appendix**

**Supplementary Table 1: Univariate analyses between vascular risk factors and neuroimaging characteristics**

| **Variables** | **Hypertension** | | **Diabetes** | | **Present drinker** | | **Present smoker** | | **Hyperlipidemia** | |
| --- | --- | --- | --- | --- | --- | --- | --- | --- | --- | --- |
|  | **r** | **P value** | **r** | **P value** | **r** | **P value** | **r** | **P value** | **r** | **P value** |
| **The presence of CMBs** | -0.006 | 0.943 | -0.07 | 0.415 | 0.02 | 0.843 | -0.02 | 0.759 | -0.07 | 0.400 |
| **The presence of lacunes** | -0.02 | 0.863 | 0.34 | <0.001 | -0.03 | 0.708 | 0.009 | 0.914 | 0.02 | 0.783 |
| **Total WMH** | 0.14 | 0.095 | 0.15 | 0.077 | 0.05 | 0.551 | 0.06 | 0.481 | -0.05 | 0.583 |
| **BG-EPVS** | 0.20 | 0.017 | 0.15 | 0.080 | -0.05 | 0.571 | -0.07 | 0.412 | 0.02 | 0.788 |
| **CSO-EPVS** | 0.14 | 0.099 | 0.15 | 0.074 | 0.004 | 0.962 | -0.07 | 0.427 | -0.02 | 0.815 |
| **Total brain volume** | 0.06 | 0.491 | 0.02 | 0.785 | 0.37 | <0.001 | 0.38 | <0.001 | 0.05 | 0.560 |
| **Gray matter volume** | 0.08 | 0.325 | 0.07 | 0.423 | 0.15 | 0.085 | 0.23 | 0.007 | 0.02 | 0.854 |
| **White matter volume** | 0.03 | 0.773 | 0.03 | 0.770 | 0.23 | 0.006 | 0.21 | 0.015 | 0.07 | 0.434 |
| **Average hippo volume** | NA | 0.999 | 0.06 | 0.520 | 0.11 | 0.214 | 0.16 | 0.052 | -0.03 | 0.732 |
|  |  |  |  |  |  |  |  |  |  |  |

Abbreviations: CMB, cerebral microbleeds; WMH, white matter hyperintensity; BG-EPVS, basal ganglia-enlarged perivascular spaces; CSO-EPVS, centrum semiovale-enlarged perivascular spaces。
